# Supplementary material for: Modeling glioblastoma heterogeneity as a dynamic network of cell states
Source: Mol Syst Biol. 2021 Sep 16;17(9):e10105. doi: 10.15252/msb.202010105 (PMC8444284; doi:10.15252/msb.202010105)
Supplement: Supplementary file 6 — Source Data for Figure 5 [file MSB-17-e10105-s004.zip › Figure5A_sourcedata/GSEA_3071/hallmarks_stateVI.GseaPreranked.1623742557031/HALLMARK_ANDROGEN_RESPONSE.html]

Details for gene set HALLMARK\_ANDROGEN\_RESPONSE[GSEA]

|  || Dataset | state63071 |
| Phenotype | NoPhenotypeAvailable |
| Upregulated in class | na\_pos |
| GeneSet | HALLMARK\_ANDROGEN\_RESPONSE |
| Enrichment Score (ES) | 0.24904351 |
| Normalized Enrichment Score (NES) | 1.3397316 |
| Nominal p-value | 0.14778325 |
| FDR q-value | 0.14561264 |
| FWER p-Value | 0.639 |
Table: GSEA Results Summary

  

Fig 1: Enrichment plot: HALLMARK\_ANDROGEN\_RESPONSE      
 Profile of the Running ES Score & Positions of GeneSet Members on the Rank Ordered List

  

| PROBE | GENE SYMBOL | GENE\_TITLE | RANK IN GENE LIST | RANK METRIC SCORE | RUNNING ES | CORE ENRICHMENT || 1 | KRT8 |  |  | 42 | 0.895 | 0.0455 | Yes |
| 2 | SMS |  |  | 88 | 0.761 | 0.0817 | Yes |
| 3 | AKAP12 |  |  | 92 | 0.743 | 0.1283 | Yes |
| 4 | UAP1 |  |  | 93 | 0.742 | 0.1757 | Yes |
| 5 | ELL2 |  |  | 156 | 0.655 | 0.2003 | Yes |
| 6 | MYL12A |  |  | 173 | 0.627 | 0.2359 | Yes |
| 7 | ADRM1 |  |  | 256 | 0.561 | 0.2490 | Yes |
| 8 | ABCC4 |  |  | 737 | 0.389 | 0.1408 | No |
| 9 | HERC3 |  |  | 754 | 0.385 | 0.1609 | No |
| 10 | ACTN1 |  |  | 845 | 0.369 | 0.1595 | No |
| 11 | PA2G4 |  |  | 986 | 0.348 | 0.1429 | No |
| 12 | PDLIM5 |  |  | 1062 | 0.335 | 0.1435 | No |
| 13 | LMAN1 |  |  | 1142 | 0.324 | 0.1423 | No |
| 14 | RRP12 |  |  | 1217 | 0.313 | 0.1418 | No |
| 15 | SORD |  |  | 1239 | 0.311 | 0.1559 | No |
| 16 | SRP19 |  |  | 1331 | 0.300 | 0.1498 | No |
| 17 | UBE2J1 |  |  | 1483 | 0.282 | 0.1259 | No |
| 18 | LIFR |  |  | 1567 | 0.272 | 0.1203 | No |
| 19 | ZBTB10 |  |  | 1941 | -0.270 | 0.0341 | No |
| 20 | SAT1 |  |  | 1952 | -0.271 | 0.0486 | No |
| 21 | ARID5B |  |  | 2156 | -0.306 | 0.0118 | No |
| 22 | IQGAP2 |  |  | 2166 | -0.308 | 0.0290 | No |
| 23 | TSC22D1 |  |  | 2193 | -0.313 | 0.0418 | No |
| 24 | RAB4A |  |  | 2289 | -0.331 | 0.0366 | No |
| 25 | B2M |  |  | 2460 | -0.369 | 0.0130 | No |
| 26 | ITGAV |  |  | 2476 | -0.371 | 0.0325 | No |
| 27 | MAF |  |  | 2633 | -0.408 | 0.0153 | No |
| 28 | SLC26A2 |  |  | 2883 | -0.489 | -0.0225 | No |
| 29 | HOMER2 |  |  | 3122 | -0.598 | -0.0503 | No |
| 30 | INPP4B |  |  | 3223 | -0.692 | -0.0338 | No |
| 31 | CDK6 |  |  | 3234 | -0.704 | 0.0083 | No |
| 32 | PMEPA1 |  |  | 3338 | -0.804 | 0.0311 | No |
| 33 | DBI |  |  | 3349 | -0.812 | 0.0801 | No |
Table: GSEA details [plain text format]

  

Fig 2: HALLMARK\_ANDROGEN\_RESPONSE: Random ES distribution      
 Gene set null distribution of ES for **HALLMARK\_ANDROGEN\_RESPONSE**
